# Discrepancy between peripapillary retinal and choroidal microvasculature and the rate of localized retinal nerve fiber layer thinning in glaucoma

**DOI:** 10.1038/s41598-023-33637-7

**Published:** 2023-04-21

**Authors:** Seung Hyen Lee, Eun Ji Lee, Tae-Woo Kim

**Affiliations:** 1 Department of Ophthalmology, Nowon Eulji University Hospital, Eulji University College of Medicine, Seoul, Korea; 2 Department of Ophthalmology, Seoul National University College of Medicine, Seoul National University Bundang Hospital, 82, Gumi-ro, 173 Beon-gil, Bundang-gu, Seongnam, 13620 Gyeonggi Korea

**Keywords:** Risk factors, Optic nerve diseases

## Abstract

This observational case series study is conducted to compare the extent of microvasculature impairment in the peripapillary retina and choroid in eyes with primary open-angle glaucoma (POAG), and to investigate the association of the discrepancy between the microvasculature impairments of each layer with the rate of progressive retinal nerve fiber layer (RNFL) thinning. A total of 88 POAG eyes with a localized RNFL defect were enrolled, including 67 eyes with and 21 eyes without choroidal microvasculature dropout (CMvD). Circumferential widths of retinal microvascular impairment (RMvI) and CMvD were measured, and eyes were classified based on the relative width of CMvD to RMvI (CMvD/RMvI ratio). The rate of RNFL thinning was determined by linear regression based on ≥ 5 serial OCT examinations. Thinner global RNFL and worse visual field mean deviation at baseline were associated with a larger circumferential width of the RMvI, whereas the presence of cold extremities, lower mean arterial pressure and thinner juxtapapillary choroid were associated with a larger circumferential width of the CMvD. The rate of global RNFL thinning was faster in eyes with larger relative CMvD width than in eyes with equal CMvD and RMvI widths and in eyes without CMvD (*P* = 0.001). Lower mean arterial pressure (*P* = 0.041), larger CMvD width (*P* = 0.046), larger CMvD/RMvI ratio (*P* = 0.041), and detection of disc hemorrhage during the follow-up (*P* = 0.013) were significant factors associated with faster global RNFL thinning. Larger CMvD width relative to RMvI width may be indicative of an increased risk of faster RNFL thinning in POAG with localized RNFL defect. Comparing the microvasculature impairment in individual layers may help predict more rapid glaucoma progression.

## Introduction

Hemodynamic factors, such as decreased ocular perfusion or vascular impairment around the optic nerve head (ONH), are considered to play a significant role in the pathogenesis of glaucoma. Two distinct vascular systems, differing according to tissue layers, are present around the ONH^1^. The superficial nerve fiber layer is supplied by the retinal circulation, including the central retinal artery (CRA), whereas deeper layers such as the choroid are supplied by the short posterior ciliary artery (SPCA), either directly or through the arterial circle of Haller and Zinn^2,3^. Because the juxtapapillary choroid is adjacent to ONH tissues and provides principal arterial input to the ONH, investigating the juxtapapillary choroidal circulation may be crucial for understanding the pathogenesis of glaucoma.

Localized microvasculature dropout (MvD) in the juxtapapillary choroid of glaucomatous eyes, as detected by optical coherence tomography angiography (OCTA), may be a pathogenic indicator of vascular impairment in glaucoma^4,5^. In addition, indocyanine green angiography has shown that a choroidal MvD (CMvD) coincided with the location of the filling defect, confirming that the area of CMvD indicated the location of decreased perfusion in the choroid^4^. The presence of CMvD has been associated with factors suggestive of systemic vascular impairment^5^, as well as more rapid glaucoma progression^6–8^. Enlargement of the CMvD has also been associated with progressive thinning of the retinal nerve fiber layer (RNFL) in glaucomatous eyes^9^. Taken together, these findings suggest that CMvD is associated with the vascular pathogenesis of glaucoma. However, it is unclear whether CMvD is a casual factor, or a secondary consequence of glaucomatous axonal damage. It is possible that decreased perfusion, as represented by CMvD, may induce ischemia and apoptotic death of retinal ganglion cells. Alternatively, the decreased metabolic needs associated with axonal damage can lead to reduced perfusion, resulting in the development of CMvD.

The present study hypothesized that the microvasculatures of the superficial retinal and choroidal layers are supplied by different systems^10^, thus this should result in a disparity in perfusion impairment in each layer during the progression of glaucoma. The mechanisms underlying the association between this disparity and glaucoma progression may provide clues to understanding the temporal relationship between choroidal microvasculature impairment and glaucomatous axonal damage. Therefore, the present study assessed the relationships between differing amounts of microvasculature impairment in the superficial retina and choroid on the clinical characteristics of patients with glaucoma, and determined the influence of these disparities on the rate of progressive RNFL thinning.

## Results

**Figure 1:**
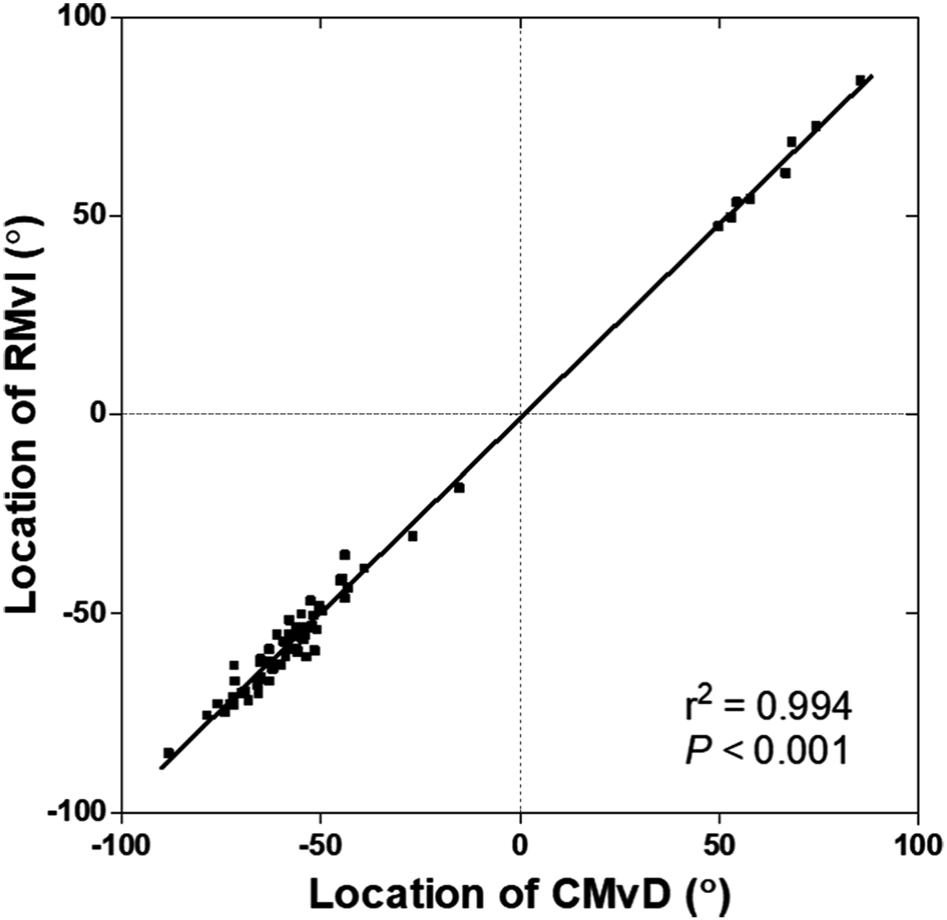
Topographic correlation between RMvI and CMvD. Positive and negative values indicate superior and inferior locations relative to the foveal-disc axis, respectively.

This study enrolled 88 eyes with primary open-angle glaucoma (POAG), all of which met the eligibility criteria. Retinal microvascular impairment (RMvI) was observed in all eyes (100%) at the location of localized RNFL defect, while CMvD was detected in 67 eyes (76.1%) at the corresponding locations. The angular location of CMvD almost exactly coincided with that of RMvI (*P* < 0.001, Fig. 1). Of the 67 eyes having CMvD, 25 had CMvD widths larger than their RMvI widths, with the difference exceeding the inter-observer variability (ratio of CMvD/RMvI widths > 1.25; CMvD + /C > R group). In the remaining 42 eyes with CMvD, the circumferential extent of CMvD was similar to that of RMvI, with the difference being within the measurement variability (ratio of CMvD/RMvI widths between 0.75 and 1.25; CMvD + /C = R group). None of the eyes with CMvD showed RMvI widths larger than the CMvD widths. The 21 eyes without CMvD were classified as the CMvD– group. Inter-observer agreements on the determination of the presence of the CMvD were excellent (κ = 0.987). The inter-observer intraclass correlation coefficients (ICC) in measuring the widths of RMvI and CMvD, and the mean juxtapapillary choroidal thickness (JPCT), were 0.983, 0.936, and 0.957, respectively.

### Characteristics of study subjects

The rate of global RNFL thinning in all included eyes was − 1.46 ± 1.55 μm/year, with a mean follow-up period of 3.4 ± 1.1 years. Table 1 shows the demographic, systemic, and ocular characteristics of the groups of patients. Cold extremities were significantly more common in patients having eyes with than without CMvD (*P* = 0.030). Patients having eyes with CMvD also had lower systemic blood pressure (BP) profiles, including lower systolic and diastolic BP, lower mean arterial pressure (MAP), and lower mean ocular perfusion pressure (MOPP; all *P* values ≤ 0.010), thinner JPCT (*P* = 0.005), and faster rate of global RNFL thinning (*P* = 0.043) than did patients having eyes without CMvD. Comparisons of eyes in the equal and larger CMvD + subgroups showed that eyes in the CMvD + /C = R group (0.75 < width of CMvD/RMvI ≤ 1.25) had worse visual field (VF) mean deviation (*P* = 0.003) and pattern standard deviation (*P* = 0.012) than did eyes in the CMvD + /C > R group (width of CMvD/RMvI > 1.25). Eyes in the CMvD + /C = R group also had a larger circumferential width of RMvI (*P* = 0.007) but a smaller circumferential width of CMvD (*P* = 0.002) than eyes in the CMvD + /C > R group. The rate of global RNFL thinning was significantly faster in the larger than in the CMvD + /C = R group (*P* = 0.022).

**Table 1:** Demographics and clinical characteristics of subjects.

Variables	CMvD– group (n = 21)	CMvD + group (n = 67)	*P* value	CMvD + group (n = 67)		
				CMvD + /C = R subgroup (n = 42)^‡^	CMvD + /C > R subgroup (n = 25)^‡^	*P* value
Demographic characteristics						
Age, years	64.8 ± 10.4	60.5 ± 10.9	0.119*	60.4 ± 10.3	60.8 ± 12.0	0.891*
Female, n (%)	16 (76.2)	42 (62.7)	0.255^†^	24 (57.1)	18 (72.0)	0.224^†^
Systemic characteristics						
Diabetes mellitus, n (%)	5 (23.8)	9 (13.4)	0.257^†^	6 (14.3)	3 (12.0)	0.791^†^
Hypertension, n (%)	5 (23.8)	10 (14.9)	0.345^†^	5 (11.9)	5 (20.0)	0.368^†^
Cold extremity, n (%)	5 (23.8)	34 (50.7)	**0.030**^†^	23 (54.8)	11 (44.0)	0.548^†^
Migraine, n (%)	6 (28.6)	22 (32.8)	0.714^†^	13 (31.0)	9 (36.0)	0.544^†^
Systolic blood pressure, mmHg	134.1 ± 14.4	121.9 ± 14.4	0.001*	121.3 ± 15.2	123.0 ± 13.2	0.633*
Diastolic blood pressure, mmHg	78.4 ± 8.7	72.2 ± 9.6	0.010*	70.9 ± 8.4	74.3 ± 11.2	0.165*
MAP, mmHg	97.0 ± 9.0	88.8 ± 10.1	0.001*	87.7 ± 9.6	90.5 ± 10.9	0.280*
MOPP, mmHg	52.9 ± 6.5	47.1 ± 7.2	0.002*	46.7 ± 7.0	47.9 ± 7.5	0.492*
Ocular characteristics						
Axial length, mm	23.71 ± 1.14	24.16 ± 1.40	0.228*	24.20 ± 1.41	24.10 ± 1.41	0.790*
Central corneal thickness, μm	528.8 ± 41.5	541.4 ± 37.2	0.190*	541.0 ± 39.8	542.0 ± 32.9	0.918*
Untreated IOP, mmHg	15.6 ± 3.0	16.1 ± 3.1	0.492*	16.2 ± 3.3	15.9 ± 2.7	0.728*
IOP at exam, mmHg	12.0 ± 2.6	12.0 ± 2.7	0.906*	11.8 ± 2.8	12.5 ± 2.4	0.309*
VF MD at baseine, dB	− 6.01 ± 4.52	− 5.41 ± 4.46	0.592*	− 6.52 ± 4.88	− 3.54 ± 2.90	0.003*
VF PSD at baseline, dB	7.34 ± 4.57	7.53 ± 4.27	0.859*	8.46 ± 4.56	5.98 ± 3.26	0.012*
Global RNFL thickness at baseline, μm	73.0 ± 13.5	76.0 ± 11.2	0.306*	74.5 ± 10.5	78.6 ± 12.0	0.142*
Circumferential width of RMvI,°	36.8 ± 20.5	42.2 ± 16.5	0.223*	46.3 ± 17.0	35.3 ± 13.3	0.007*
Circumferential width of CMvD,°	n/a	51.7 ± 20.7	n/a	46.7 ± 17.8	62.5 ± 21.9	0.002*
Ratio of CMvD width to RMvI width	n/a	n/a	n/a	1.01 ± 0.05	1.86 ± 0.50	** < 0.001**
Detection of DH during follow-up, n (%)	6 (28.6)	20 (29.9)	0.911^†^	10 (23.8)	10 (40.0)	0.161^†^
Mean JPCT, μm	136.2 ± 37.2	110.5 ± 35.6	0.005*	109.5 ± 38.8	112.0 ± 30.0	0.785*
Rate of global RNFL thinning, μm/year	− 0.85 ± 0.89	− 1.64 ± 1.68	0.043*	− 1.20 ± 0.91	− 2.38 ± 2.33	0.022*
Duration of follow up, year	3.6 ± 1.7	3.4 ± 0.9	0.364*	3.5 ± 0.8	3.1 ± 0.9	0.158*

Values area mean ± standard deviation unless otherwise indicated. Values with statistical significance are shown in bold.

*CMvD* choroidal microvasculature dropout, *MAP* mean arterial pressure, *MOPP* mean ocular perfusion pressure, *IOP* intraocular pressure, *VF* visual field, *MD* mean deviation, *PSD* pattern standard deviation, *RNFL* retinal nerve fiber layer, *RMvI* retinal microvascular impairment, *n/a* not applicable, *DH* disc hemorrhage, *JPCT* juxtapapillary choroidal thickness.

*Comparisons were performed using independent samples *t*-test.

^†^Comparisons were performed using Pearson’s *chi*-square test.

^‡^Ratio of the circumferential width of CMvD to that of the RMvI, between 0.75 and 1.25 and > 1.25 were defined as CMvD + /C = R and CMvD + /C > R, respectively.

### Factors associated with the circumferential widths of the RMvI and CMvD

Univariable and multivariable analyses showed that the circumferential width of the RMvI was significantly associated with worse VF mean deviation (*P* < 0.001) and reduced global RNFL thickness (*P* = 0.013), whereas the circumferential width of the CMvD was significantly associated with the presence of cold extremities (*P* = 0.019), lower MAP (*P* = 0.030) and thinner JPCT (*P* = 0.015; Table 2).

**Table 2:** Factors associated with the circumferential width of RMvI and CMvD in all POAG Eyes (n = 88).

Variables	Circumferential width of RMvI					Circumferential width of CMvD				
	Univariable		Multivariable analysis			Univariable		Multivariable analysis		
	Βeta	*P*	Βeta	*P*	VIF	Βeta	*P*	Βeta	*P*	VIF
Demographic characteristics										
Age, per 1 year older	0.052	0.299				− 0.156	0.587			
Gender, female	− 1.796	0.653				1.124	0.864			
Systemic characteristics										
Diabetes	1.602	0.757				− 4.702	0.580			
Hypertension	− 3.233	0.520				− 6.105	0.459			
Cold extremities	5.675	0.134				13.282	**0.031**	**13.919**	**0.019**	1.008
Migraine	1.892	0.641				0.242	0.971			
MAP, per 1 mmHg higher	− 0.130	0.475				− 0.557	**0.061**	− **0.620**	**0.030**	1.019
Ocular characteristics										
Axial length, per 1 mm longer	− 0.489	0.731				− 0.447	0.851			
Central corneal thickness, per 1 μm thicker	− 0.039	0.430				0.109	0.179			
Untreated IOP, per 1 mmHg higher	0.611	0.326				1.343	0.187			
IOP at exam, per 1 mmHg higher	0.120	0.869				1.538	0.196			
VF MD at baseline, per 1 dB higher	− **2.181**	** < 0.001**	− **1.758**	** < 0.001**	1.234	− 0.977	0.161			
Global RNFL thickness at baseline, per 1 μm thicker	− **0.658**	** < 0.001**	− **0.368**	**0.013**	1.234	− 0.075	0.778			
Detection of DH during follow-up	− 1.846	0.656				1.605	0.814			
Mean JPCT, per 1 μm thicker	− 0.002	0.973				− **0.156**	**0.058**	− **0.194**	**0.015**	1.024

Variables with *P* < 0.1 in the univariable analysis were included in the multivariable analysis.

Values with statistical significance are shown in bold.

*RMvI* retinal microvascular impairment, *CMvD* choroidal microvascular dropout, *VIF* variance inflation factor, *MAP* mean arterial pressure, *IOP* intraocular pressure, *VF* visual field, *MD* mean deviation, *RNFL* retinal nerve fiber layer, *DH* disc hemorrhage, *JPCT* juxtapapillary choroidal thickness.

### Associations of the presence/absence and circumferential width of the CMvD with the rate of RNFL thinning

**Figure 2:**
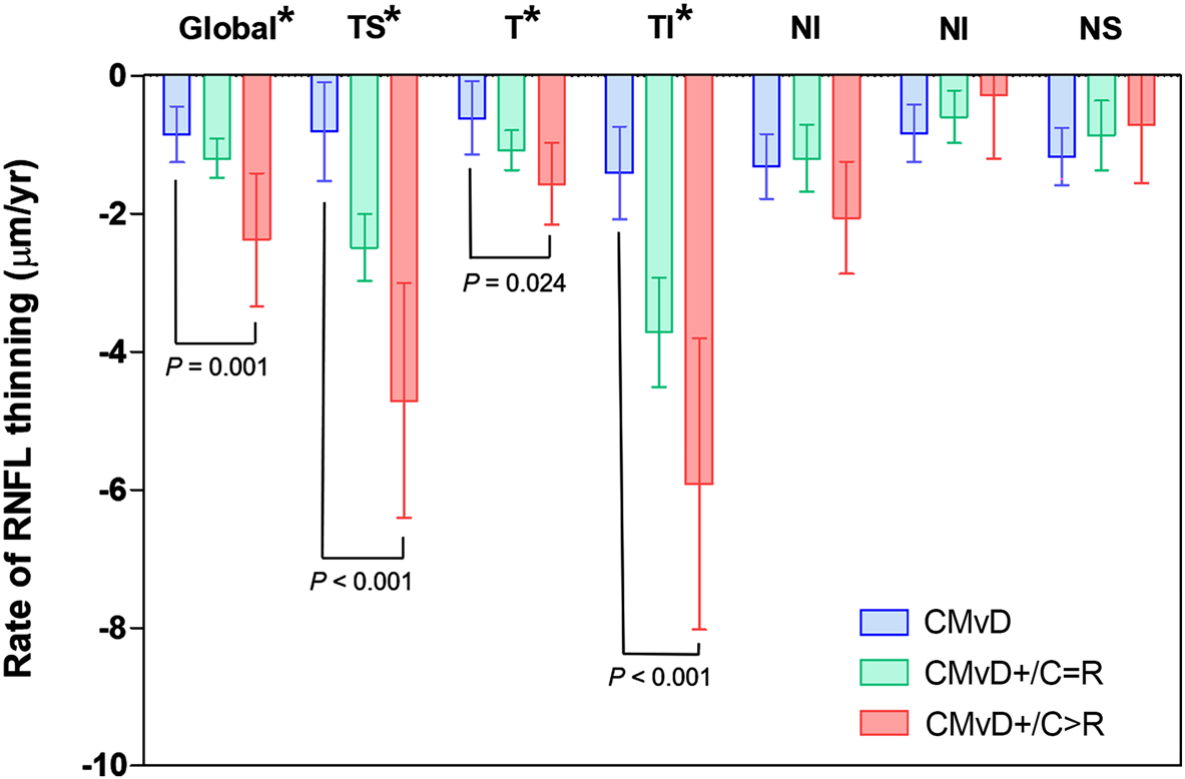
Comparison of the rate of retinal nerve fiber layer (RNFL) thinning globally and in six circumpapillary sectors. Asterisks indicate statistically significant differences between groups. Error bars indicate 95% confidence intervals.

Table 3 and Fig. 2 show the rates of RNFL thinning as a function of the presence/absence and circumferential width of the CMvD. The rates of global RNFL thinning (*P* = 0.001) and thinning in the TS (*P* < 0.001), T (*P* = 0.024) and TI (*P* < 0.001) sectors differed significantly in the CMvD–, CMvD + /C > R, and CMvD + /C = R groups. The rate of global RNFL thinning was faster in the CMvD + /C > R group (− 2.38 ± 2.33 μm/year) than in the CMvD + /C = R (− 1.20 ± 0.91 μm/year) and CMvD– (− 0.85 ± 0.89 μm/year; *P* = 0.001) groups. Similarly, the rates of RNFL thinning were greatest in the CMvD + /C > R group, followed by the CMvD + /C = R and CMvD– groups in the TS (− 4.71 ± 4.12 *vs*. − 2.49 ± 1.54 *vs*. − 0.81 ± 1.57 μm/year, respectively; *P* < 0.001) and TI (− 5.92 ± 5.11 *vs*. − 3.72 ± 2.52 *vs*. − 1.41 ± 1.48 μm/year, respectively; *P* < 0.001) sectors. In the T sector, the rate of thinning differed significantly only in the CMvD– and CMvD + /C > R groups (− 0.62 ± 1.17 *vs.* − 1.57 ± 1.42 μm/year, respectively;* P* = 0.024).

**Table 3:** Comparison of rate of circumpapillary retinal nerve fiber layer thinning per year among groups.

Rate of RNFL thinning (μm/yr)	No CMvD– group (n = 21)	CMvD + /C = R group* (n = 42)	CMvD + /C > R group* (n = 25)	*P* value	Post-hoc
Global	− 0.85 ± 0.89	− 1.20 ± 0.91	− 2.38 ± 2.33	**0.001**	CMvD–, CMvD + /C = R > CMvD + /C > R
TS	− 0.81 ± 1.57	− 2.49 ± 1.54	− 4.71 ± 4.12	** < 0.001**	CMvD– > CMvD + /C = R > CMvD + /C > R
T	− 0.62 ± 1.17	− 1.08 ± 0.93	− 1.57 ± 1.42	**0.024**	CMvD– > CMvD + /C > R
TI	− 1.41 ± 1.48	− 3.72 ± 2.52	− 5.92 ± 5.11	** < 0.001**	CMvD– > CMvD + /C = R > CMvD + /C > R
NI	− 1.32 ± 1.03	− 1.20 ± 1.55	− 2.06 ± 1.97	0.091	
N	− 0.84 ± 0.92	− 0.60 ± 1.23	− 0.29 ± 2.20	0.466	
NS	− 1.18 ± 0.91	− 0.87 ± 1.62	− 0.71 ± 2.04	0.608	

Values area mean ± standard deviation. Values with statistical significance are shown in bold.

Comparison was performed using 1-way analysis of variance with post hoc Sidak multiple comparison testing.

Values that were significant are shown in bold.

*RNFL* retinal nerve fiber layer, *CMvD* choroidal microvascular dropout, *TS* temporo-superior, *T* temporal, *TI* temporo-inferior, *NI* naso-inferior, *I* inferior, *NS* naso-superior.

*Ratio of the circumferential width of CMvD to that of the RMvI, between 0.75 and 1.25, and > 1.25 were defined as CMvD + /C = R and CMvD + /C > R, respectively.

### Factors associated with the rate of global RNFL thinning

**Figure 3:**
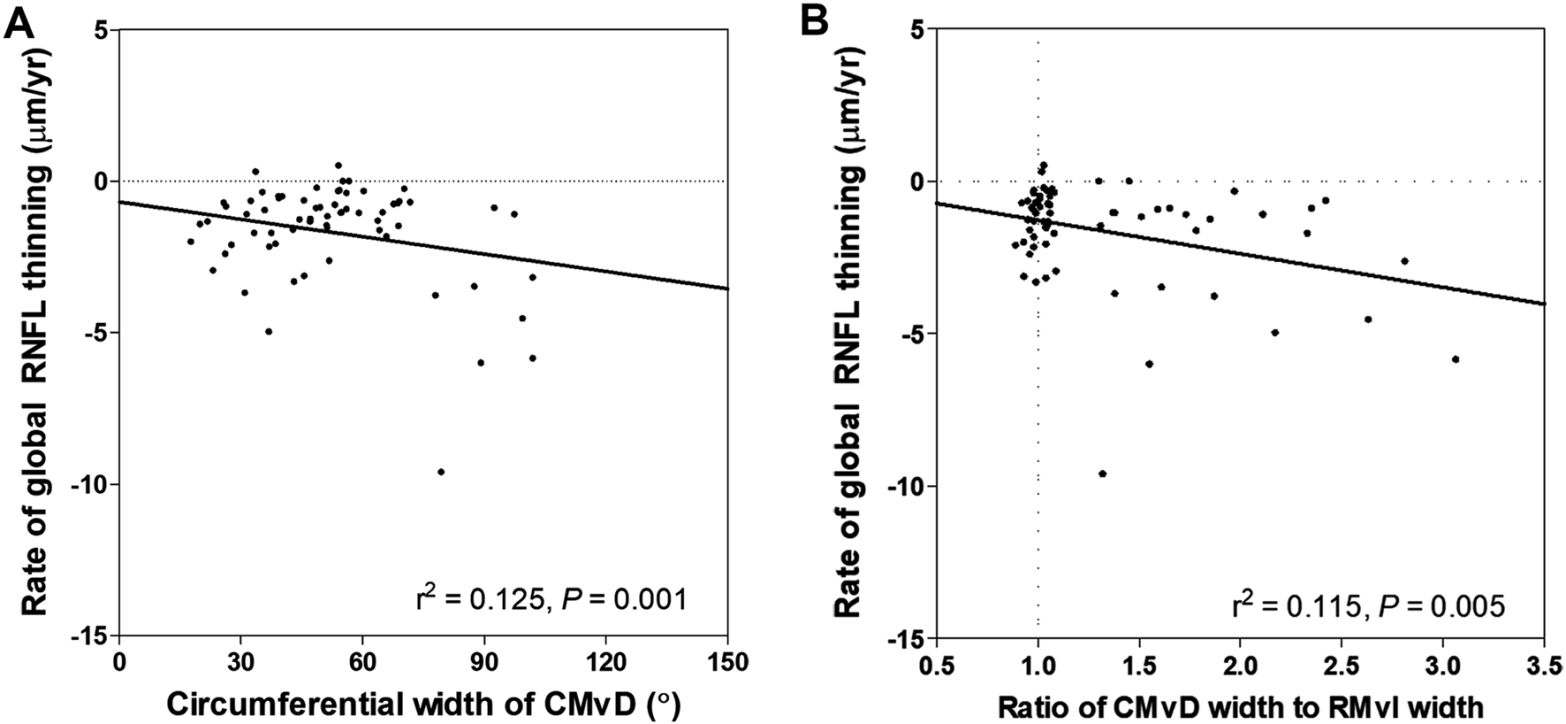
Correlations between the rate of global RNFL thinning and (**A**) the circumferential width of the CMvD and (**B**) the ratio of CMvD width to RMvI width.

Univariable regression analysis showed that lower MAP (*P* = 0.048), larger circumferential width of CMvD (*P* = 0.001), larger ratio of CMvD width to RMvI width (*P* = 0.005), and the detection disc hemorrhage during follow-up (*P* = 0.004) were significantly associated with a faster rate of global RNFL thinning (Table 4). Similarly, multivariable analysis showed that the rate of global RNFL thinning was associated with lower MAP (*P* = 0.041), larger width of CMvD (*P* = 0.046), larger ratio of CMvD width to RMvI width (*P* = 0.041), and disc hemorrhage (*P* = 0.013) (Table 4, Fig. 3).

**Table 4:** Factors associated with rate of global retinal nerve fiber layer thinning in all POAG eyes (n = 88).

Variables	Univariable analysis		Multivariable analysis		
	Βeta	*P*	Βeta	*P*	VIF
Demographic characteristics					
Age, per 1 year older	− 0.010	0.519			
Gender, female	− 0.290	0.412			
Systemic characteristics					
Diabetes	0.717	0.116			
Hypertension	0.024	0.958			
Cold extremities	0.061	0.858			
Migraine	− 0.125	0.730			
MAP, per 1 mmHg higher	0.032	**0.048**	0.038	**0.041**	1.038
Ocular characteristics					
Axial length, per 1 mm longer	0.089	0.490			
Central corneal thickness, per 1 μm thicker	− 0.003	0.440			
Untreated IOP, per 1 mmHg higher	− 0.055	0.321			
IOP at exam, per 1 mmHg higher	− 0.054	0.403			
VF MD at baseline, per 1 dB higher	− 0.056	0.138			
Global RNFL thickness at baseline, per 1 μm thicker	− 0.020	0.159			
Circumferential width of RMvI, per 1° larger	− 0.006	0.512			
Circumferential width of CMvD, per 1° larger	− 0.019	**0.001**	− 0.019	**0.046**	1.180
Ratio of CMvD width to RMvI width	− 1.101	**0.005**	− 0.811	**0.041**	1.229
Detection of DH during follow-up	− 1.031	**0.004**	− 1.017	**0.013**	1.021
Mean JPCT, per 1 μm thicker	0.003	0.568			

Variables with *P* < 0.1 in the univariable analysis were included in the multivariable model.

Values with statistical significance are shown in boldface.

*VIF* variance inflation factor, *MAP* mean arterial pressure, *IOP* intraocular pressure, *VF* visual field, *MD* mean deviation, *RNFL* retinal nerve fiber layer, *RMvI* retinal microvascular impairment, *CMvD* choroidal microvasculature dropout, *DH* disc hemorrhage, *JPCT* juxtapapillary choroidal thickness.

### Representative cases

**Figure 4:**
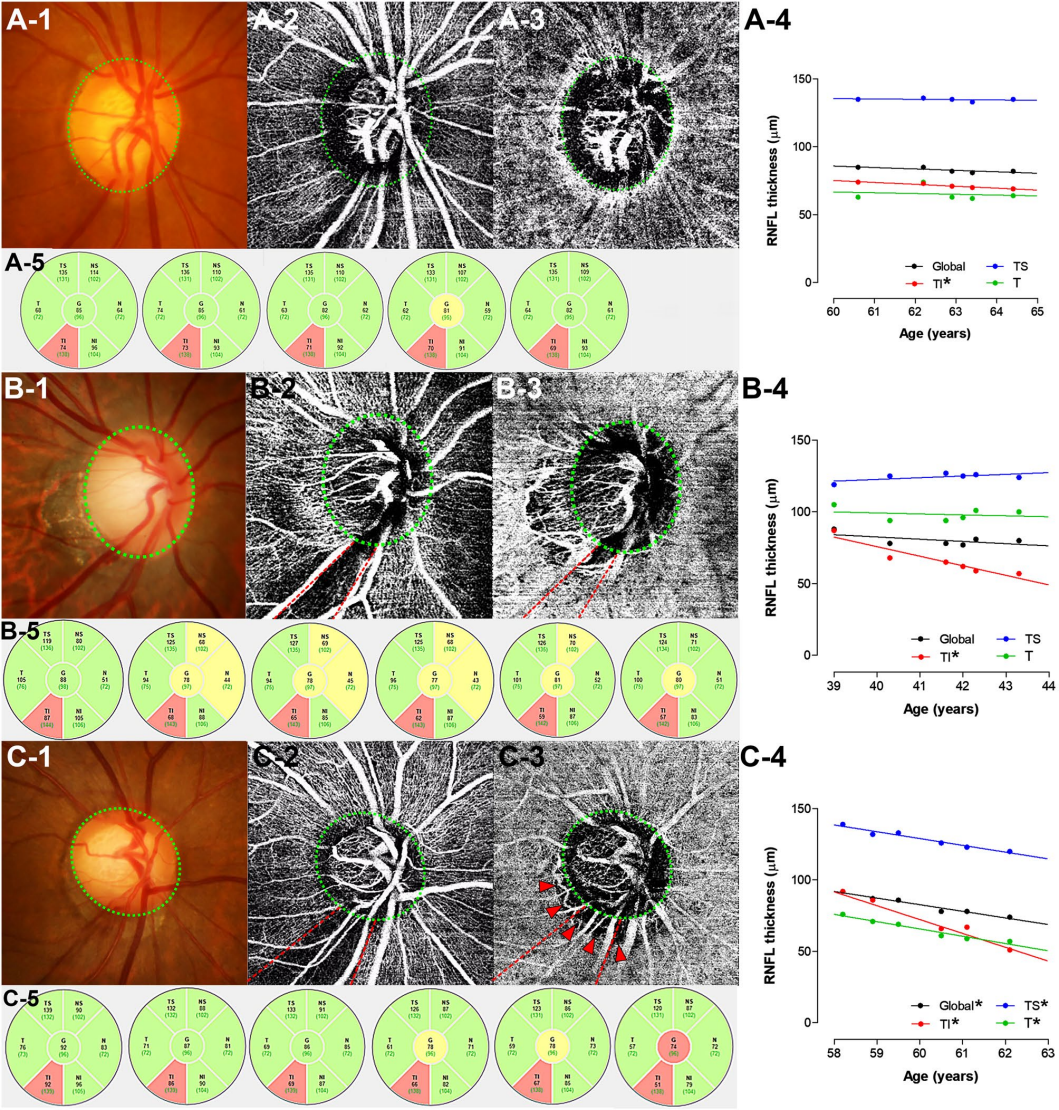
Representative eyes with primary open-angle glaucoma (**A**) without a CMvD, (**B**) with a CMvD width similar to the RMvI width, and (**C**) with a CMvD width larger than the RMvI width. (**A-1**,**B-1**,**C-1**) Color disc photographs, (**A-2**,**B-2**,**C-2**) En face OCTA images of the retinal and (**A-3**,**B-3**,**C-3**) choroidal layers. *Green dashed ellipses* indicate margins of the optic discs. *Red dashed lines and arrowheads* indicate borders of RMvIs and CMvDs, respectively. (**A-4**,**B-4**,**C-4**) Graphs showing the rate of RNFL thinning globally and in the TS, T, and TI circumpapillary sectors. Asterisks indicate the rates of RNFL thinning with statistical significance. The rates of RNFL thinning were relatively low in all sectors of eyes without CMvD (**A-4**), but were significantly higher in eyes with CMvD (**B-4**,**C-4**). Note that the eye with CMvD width similar to RMvI width showed significantly rapid RNFL thinning at the location of the CMvD and RMvI (**B-4**), whereas the eye with CMvD width larger than RMvI width also showed rapidly progressive RNFL thinning outside the RMvI (**C-4**). (**A-5**,**B-5**,**C-5**) Serial OCT sectoral RNFL thickness map. Numbers represent RNFL thickness in micrometers. *TS* temporal-superior, *T* temporal, *TI* temporo-inferior.

Figure 4 presents three eyes with POAG, one each in the CMvD–, CMvD +/C = R, and CMvD +/C > R group. The eye without CMvD showed relatively slow rates of RNFL thinning, both globally and in the TS, T and TI sectors, compared with the other two CMvD + eyes. The eye having a similar circumferential extent of CMvD and RMvI showed faster RNFL thinning at the location of CMvD in the TI sector, but thinning of RNFL thickness (RNFLT) in global and other sectors was not evident. In contrast, the eye having a larger ratio of CMvD width to RMvI width showed rapid RNFL thinning globally and in the TS, T and TI sectors. Interestingly, the rate of progressive RNFL thinning was significant in the T sector, a location at which the CMvD width exceeded the RMvI width.

## Discussion

This study showed a discrepancy between the extents of microvasculature impairment in the retinal and choroidal layers among eyes with POAG. The rate of RNFL thinning was greater in eyes with than without CMvD. Moreover, among the CMvD+ eyes, those having a CMvD extent larger than the RMvI extent showed more rapid RNFL thinning than those with a CMvD extent equal to the RMvI extent. The present findings suggest that the disparity between choroidal and superficial microvasculature impairment can be an indicator of rapid progression of glaucoma.

OCTA has shown impairment of the peripapillary microvasculature in both the retinal^11,12^ and choroidal^4,5^ layers of eyes with POAG. Systemic hypoperfusion may reduce ocular perfusion via both the CRA and SPCA, resulting in a general reduction of retinal and choroidal microvascular signals in OCTA. In contrast, glaucomatous damage frequently results in a localized RNFL defect, accompanied by a characteristic localized reduction in the retinal microvasculature (i.e., RMvI)^13^. Such an RMvI is considered a consequence of structural loss of RPC at the location of the thinned RNFL, thus coinciding with the pattern of the RNFL defect^14^. On the other hand, the reduction in prelaminar axons supplied by the PCA system may reduce metabolic need, leading to localized damage that can also cause secondary loss of the choroidal microvasculature (i.e., CMvD)^15^. Alternatively, however, the CMvD may be an indicator of primary perfusion impairment and a predictor of glaucoma progression^6,7,16^. It is therefore unclear whether the CMvD is a primary cause or secondary consequence of glaucomatous damage.

Similar to previous findings^4,5^, the present study also showed that the presence of CMvD was significantly associated with several vascular risk factors, including cold extremities, low systemic BP profiles, and thinner mean JPCT. CMvD may be a representative vascular risk factor, and may act as a predictor of glaucomatous damage.

In addition to the distinct characteristics associated with the presence of CMvD, more rapid RNFL thinning in the present study was observed in eyes in which the CMvD width was larger than the RMvI width than in eyes with equal widths. In contrast, none of the eyes in the present study had an RMvI width that was larger than the CMvD width. Because peripapillary choroidal circulation is closely linked to ONH perfusion, CMvD has been considered a marker of compromised choroidal microvasculature in relation to glaucoma. The finding, that eyes with higher CMvD/ RMvI ratios showed more rapid RNFL thinning, both globally and in all temporal sectors, suggests that progressive axonal loss may be ongoing, even at locations at which the RMvI is not yet reduced. Because active transport along extended axons requires significant metabolic demands^17^, the axons in the prelaminar and laminar portions are more vulnerable to hypoperfusion. Insufficient blood supply at this level not only inhibits axonal transport but can also produce ischemic insult to the axons, leading to apoptosis of retinal ganglion cells. CMvD enlargement has been associated with progressive structural^9^ and functional^18^ damage in eyes with POAG. These findings, along with the results of the present study, suggest that the perfusion was first reduced at the location of CMvD, subsequently damaging the RNFL and resulting in widening of the RMvI. Vessel defects in the superficial layer may be an overall ramification of glaucomatous damage, whereas defects in the deep layer are more likely to be affected by factors related to ocular microcirculation^19^. The results of the present study showed that RMvI width was closely related to the current status of glaucomatous damage, whereas CMvD width was highly associated with future progression.

It is again unclear whether a CMvD is a cause or consequence of glaucomatous damage. A CMvD occurring secondary to glaucomatous injury would be expected to develop later than RMvI, or at least be smaller in size. However, despite the finding that some eyes had RMvI but not CMvD (CMvD– group), few eyes had CMvD widths smaller than RMvI widths when they once showed a CMvD (CMvD + group). In contrast, eyes with CMvD width equal to RMvI width showed worse VF damage and larger RMvI than eyes with larger relative CMvD widths. These eyes may have undergone glaucomatous progression before the width of the RMvI reached the boundaries of the CMvD.

In the present study, intraocular pressure (IOP) was not associated with the rate of global RNFL thinning. In contrast, previous studies reported that IOP was the most important risk factor in the progression of glaucoma^20–22^. This discrepancy between the findings may be attributable to most of the patients in our study having untreated IOP within the normal range. On the other hand, lower MAP was found to be significantly associated with rapid RNFL thinning in the present study. Although increased IOP is considered the most important risk factor for the development^23^ and progression^20,24,25^ of glaucoma, compromised ocular blood flow^26–30^ or decreased perfusion in the retina and choroid^31–36^ have also been associated with glaucoma. Lower MAP has been shown to affect the rates of structural progression in glaucoma when IOP is well-controlled^37^. Taken together, these findings suggest that lower arterial pressure may be a potentially modifiable risk factor for glaucoma, especially in the eyes with lower IOP.

In agreement with previous results^7,38^, the present study found that disc hemorrhage was an independent factor for rapid RNFL thinning. However, neither the presence of CMvD nor its width was associated with episodes of disc hemorrhage. The pathogenic mechanism of disc hemorrhage and its association with glaucomatous progression are not clear. However, the present results suggest that disc hemorrhage may not be associated with peripapillary vascular impairment, as represented by CMvD.

The present study had several limitations. First, it only included POAG eyes with a distinct focal RNFL defect, thereby excluding eyes with multiple RNFL defects or advanced glaucoma. Although the present results may not be applicable to these latter eyes, we believe that our findings may be helpful in understanding the influence of relative width of CMvD on the rate of RNFL thinning during relatively early stages of glaucoma. Second, retinal vessel signals observed in the choroidal OCTA images could have hampered a precise evaluation of the boundary of CMvD. However, the effects of these projection artifacts were minimized by excluding images with ambiguous margins and by evaluation of images by multiple observers. Third, this study included only Koreans, with a high proportion having normal tension glaucoma. This may have introduced a selection bias, because patients with normal tension glaucoma are generally more affected by the vasculature than patients with high tension glaucoma. Similar studies in patients with different races and ethnicities are needed to confirm the findings of the present study. Finally, the present study did not consider the structure of peripapillary tissue (i.e., β- and γ-zone parapapillary atrophy). The pathogenic mechanism of CMvD depends on the accompanying peripapillary microstructure, thus altering its clinical significance^39^. However, the present study excluded highly myopic eyes with axial length (AXL) > 26 mm and eyes with tilted optic discs, which are likely to have large γ-zones. All subjects likely had β-zone parapapillary atrophy, suggesting a uniform pathogenesis of CMvD in these patients.

In conclusion, the present study found that peripapillary microvascular impairment did not coincide in the retinal and choroidal layers of glaucomatous eyes with localized RNFL defect. Not only having differed clinical characteristics, association with progressive RNFL thinning also differed according to the relative extent of CMvD to that of RMvI. Discrepancies in microvascular impairment in individual layers may be a biomarker for glaucoma progression.

## Materials and methods

The present study included patients with POAG who were enrolled in the Investigating Glaucoma Progression Study (IGPS), an ongoing prospective study started in August 2011 at the Glaucoma Clinic of Seoul National University Bundang Hospital. The protocol of the present study was approved by the Institutional Review Board of Seoul National University Bundang Hospital (No.: B-2210-786-104), and conformed to the tenets of the Declaration of Helsinki. All included subjects provided written informed consent.

### Study subjects

A database of subjects included in the IGPS between July 2016 and January 2022 was reviewed. All subjects who were enrolled in the IGPS underwent a complete ophthalmic examination, including visual acuity assessment, refraction, slit-lamp biomicroscopy, gonioscopy, Goldmann applanation tonometry, and dilated stereoscopic examination of the optic disc. They also underwent measurements of corneal curvature (KR-1800, Topcon, Tokyo, Japan), central corneal thickness (Orbscan II, Bausch & Lomb Surgical, Rochester, NY, USA) and AXL (IOL Master version 5, Carl Zeiss Meditec, Dublin, CA, USA); stereoscopic disc photography and red-free fundus photography (EOS D60 digital camera, Canon, Utsunomiya-shi, Tochigi-ken, Japan); measurements of circumpapillary RNFLT and scanning of the optic disc using spectral-domain OCT (SD-OCT; Spectralis OCT, Heidelberg Engineering, Heidelberg, Germany); scanning of the optic disc using OCTA (DRI-OCT Triton, Topcon); and standard automated perimetry (24-2 Swedish interactive threshold algorithm and Humphrey Field Analyzer II 750, Carl Zeiss Meditec).

Subjects included in the IPGS were required to have a best-corrected visual acuity > 20/40. To be included in the present study, subjects were required to have an AXL < 26.0 mm and not to have a tilted disc (i.e., a ratio of longest to shortest optic disc diameter > 1.3)^40,41^. Eyes with a history of intraocular surgery other than cataract or glaucoma surgery were excluded. Although a history of cataract or glaucoma surgery prior to the baseline examination was not a criterion for exclusion, patients who underwent such procedures during the study period were excluded because these procedures could affect the RNFLT. Eyes with a history of ocular trauma or uveitis were also excluded, as were patients with other intraocular diseases (e.g., age-related macular degeneration, diabetic retinopathy, or retinal vessel occlusion) or neurological diseases (e.g., pituitary tumors) that could cause VF defects. Patients with any abnormalities in the circumpapillary region that affected the scan circle in which RNFLT was measured by OCT were also excluded from this study.

Patients included in the present study were required to be diagnosed with POAG and have a localized RNFL defect that was clearly visible by red-free fundus photography. This criterion enabled a comparison of the extents of microvascular deteriorations in the retinal and choroidal layers at the location with distinct RNFL damage. Eyes were also required to have undergone OCTA imaging of the optic nerve at baseline and to have undergone at least five serial circumpapillary RNFLT measurements by SD-OCT at intervals of 6 months to 1 year, with the interval based on the expected rate of progression in individual eyes. If eyes showed progressive RNFL thinning based on RNFLT measurement and an assessment of the circumpapillary B-scan images, next SD-OCT was performed prior to their regularly scheduled examination. When both eyes of a subject were eligible for inclusion, one eye was chosen randomly for inclusion in the data analysis.

POAG was defined as the presence of an open iridocorneal angle, an abnormal glaucomatous optic disc (diffuse or focal thinning of the neuroretinal rim or a splinter hemorrhage) and associated VF defects without other ocular diseases or conditions that might cause VF abnormalities. A glaucomatous VF defect was defined as (1) values outside the normal limits in the glaucoma hemifield test; (2) three abnormal points, each with a < 5% probability of being normal, and one point with a pattern deviation of *P* < 1%; or (3) a pattern standard deviation of *P* < 5%. These VF defects were confirmed on two consecutive reliable tests (fixation loss rate ≤ 20% and false-positive and false-negative error rates ≤ 25%).

Untreated IOP was defined as the mean of at least two measurements made before receiving IOP-lowering treatment. IOP at exam was defined as that measured at the time OCTA images were obtained. Disc hemorrhage was defined as an isolated hemorrhage seen on the optic disc or peripapillary area connected to the disc rim^42^. Disc hemorrhage was recorded at every visit during the follow-up period, and the eyes showing disc hemorrhage at least once was considered having history of disc hemorrhage.

Systolic and diastolic BP were measured at the time of OCTA. The MAP was calculated using the formula MAP = diastolic BP + 1/3 (systolic BP-diastolic BP), and MOPP was calculated using the formula MOPP = 2/3 MAP-IOP.

### OCTA assessment of retinal and choroidal microvasculature impairments

At baseline, the optic nerve and peripapillary area were imaged using a commercially available swept-source OCTA device (DRI-OCT Triton, Topcon) with a central wavelength of 1050 nm, an acquisition speed of 100,000 A-scans per second, and axial and transversal resolutions in tissue of 7 mm and 20 mm, respectively. Scans were obtained from 4.5-mm × 4.5-mm cubic areas, with each cube consisting of 320 clusters of four repeated B-scans centered on the optic disc.

En-face projections of volumetric scans allowed visualization of the structural and vascular details of various user-defined retinal and choroidal layers, which were generated based on the automated layer segmentation performed by the OCTA instrument software. The retinal and choroidal microvasculature was evaluated on en-face OCTA images of the superficial retinal and choroidal layers, respectively, while masking the position of microvascular defect in each layer. The superficial layer included the RNFL, ganglion cell layer, and inner plexiform layer (i.e., from the internal limiting membrane to the outer border of the inner plexiform layer). In contrast, the en-face images of the deep layer were derived from an en-face slab that extended from the retinal pigment epithelium to the outer border of the sclera, which included the choroid. The RMvI as determined by OCTA was defined as a region of decreased vascularity in the retinal layer, as indicated by a clearly demarcated darker area with decreased capillary density relative to the adjacent area^13^. CMvD was defined as a focal sectoral capillary dropout with no visible microvascular network in the choroidal-layer en-face images, as described previously^4,43^. A circumferential width of the area with capillary dropout exceeding half a clock hour of the disc circumference was considered a disruption of the microvascular network and deemed a CMvD^43,44^.

RMvIs and CMvDs were identified independently by two observers (S.H.L. and E.J.L.), who were masked to the clinical characteristics of the study subjects. Discrepancies between these two observers were resolved by a third observer (T.-W.K.). If the OCTA images of an eye were of poor quality (e.g., blurred images that hampered the delineation of the vascular defect), that eye was excluded from the analysis.

### Determination of the circumferential extent and angular location of the RMvI and CMvD

**Figure 5:**
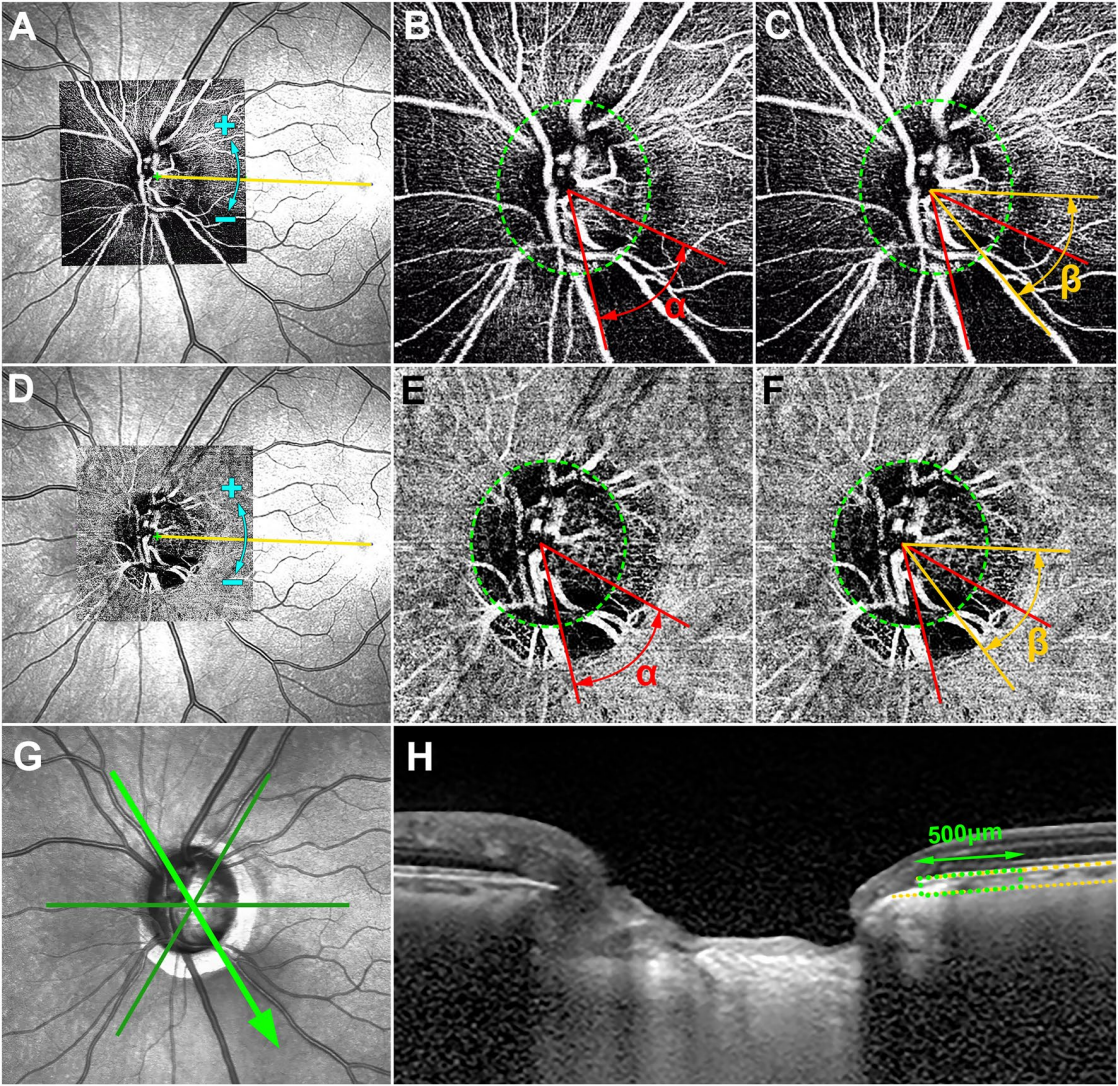
Determination of the circumferential extent and location of retinal microvascular impairment (RMvI) and choroidal microvasculature dropout (CMvD) and juxtapapillary choroidal thickness (JPCT). En-face OCTA images of the retinal (**A**–**C**) and choroidal (**D**–**F**) layers were superimposed and manually aligned on the infrared fundus photographs to determine the foveal-disc axes (*yellow lines*, **A**,**D**). Positive and negative angles indicated the locations that were superior and inferior relative to the foveal–disc axis, respectively (**A**,**D**). *Green dashed ellipses* indicate optic disc margins (**B**,**C**,**E**,**F**). The circumferential widths of RMvI and CMvD were measured as the angular distances between the two points at which the borders of the RMvI or CMvD area met the clinical optic disc margins (α, **B**,**E**). The locations of the RMvIs and CMvDs were defined as the angular distances between the foveal-disc axis and the midpoint dividing the *red lines*, indicating the circumference of the RMvI or CMvD (β, **C**,**F**). Three radial B-scans were selected for the evaluation of the JPCT (*green lines*, **G**). The image in (**H**) shows one of the radial B-scans that was obtained along superonasal to inferotemporal meridian (*green arrow*, **G**). *Yellow dotted lines* indicate the upper and lower margins of the parapapillary choroid (**H**), and the area of the choroidal tissue within 500 μm from the border tissue of Elschnig (*green dotted square*) was measured using the built-in drawing tool of Spectralis viewer software. The mean JPCT was calculated by dividing the area by 500 μm.

The circumferential width and angular location of the RMvI and CMvD were measured as the angular extent (α) and as the angular deviations of the midpoints of the RMvIs and CMvDs relative to the foveal-disc axis (β), respectively (Fig. 5). Positive and negative values of the angular locations of the RMvI and CMvD indicated the locations that were superior and inferior relative to the foveal–disc axis, respectively. These measurements were made by first identifying the two points at which the borders of the RMvI and CMvD areas met the optic disc margin. Lines connecting these points and the disc center were drawn and used to measure the circumferential width and location of the RMvI and CMvD. The OCTA images were superimposed on and manually aligned with the infrared fundus images obtained by SD-OCT circumpapillary scanning, and the foveal-disc axes in OCTA images were determined using commercially available software (Photoshop CC; Adobe Systems, Mountain View, CA, USA).

The circumferential widths and angular locations of the RMvIs and CMvDs were determined by two independent observers (S.H.L. and E.J.L.) masked to the clinical characteristics of the study subjects. The mean of the measurements made by the two observers was used for analysis.

Eyes were divided into groups based on the presence/absence of CMvD (i.e., the CMvD + and CMvD– groups), with the former further subdivided based on the relative width of the CMvD to that of the RMvI (i.e. CMvD + /C > R and CMvD + /C = R subgroups). Significant differences between the circumferential extents of the CMvD and RMvI were determined based on the inter-observer variability of the ratio of CMvD/RMvI widths ((i.e. greater than two-fold standard deviation [SD] of interobserver measurement difference).

### Determination of the rate of retinal nerve fiber layer thinning

Circumpapillary RNFLT was measured using a circular scanning protocol of the Spectralis OCT system, as described previously^45^. The diameter of the scan circle spanned 12°, with the diameter in millimeters depending on the AXL. The Spectralis OCT system divides the circumpapillary scanning circle into six sectors based on the foveal-disc axis: temporal (T, 316°–45°), temporal-superior (TS, 46°–90°), nasal-superior (NS, 91°–135°), nasal (N, 136°–225°), nasal-inferior (NI, 226°–270°), and temporal-inferior (TI, 271°–315°). The global RNFLT and the RNFLT in each of the six sectors were recorded for analysis. The rates of RNFL thinning, in microns per year, were determined by linear regression of serial OCT measurements of global RNFLT and RFNLT in the six sectors. Eyes with poor-quality B-scan images, in which the boundaries of the RNFL could not be identified (i.e., quality score < 15), were excluded.

### Evaluation of juxtapapillary choroidal thickness

The JPCT was measured at six juxtapapillary locations (temporal, temporal-superior, temporal-inferior, nasal-inferior, nasal, and nasal-superior) on three radial B-scan images of the ONH, using the built-in drawing tool of the Spectralis viewer software (Heidelberg Eye Explorer software version 1.7.0.0; Heidelberg Engineering). The corneal curvature of each eye was entered into the Spectralis OCT system prior to OCT scanning to compensate for any magnification error. Eyes with poor-quality B-scan images, in which the boundaries of the RNFL could not be identified (i.e., quality score < 15), were excluded.

The JPCT was measured in each radial scan, as described previously^46,47^. Briefly, the area of the choroidal tissue within 500 μm from the border tissue of Elschnig was measured, with the JPCT calculated by dividing the choroidal area by 500 μm (Fig. 5). The measurements obtained at the abovementioned six locations were averaged and defined as the mean JPCT. Measurements were made, and mean JPCTs calculated, by two experienced observers (S.H.L. and E.J.L.) who were blinded to subjects’ clinical characteristics, with the average of these two mean JCPTs included for analysis.

### Statistical analysis

The inter-observer agreements for determining the presence of CMvD were assessed using kappa statistics (κ value), and those for the measurements of the circumferential extent and location of the RMvI and CMvD, and for the measurements of JPCT, were assessed using the ICC. Between-group comparisons of continuous and categorical variables were performed using Student’s *t*-tests and chi-squared tests, respectively. Rates of RNFL thinning among more than two groups were compared by one-way analysis of variance (ANOVA) with post hoc Sidak tests. Factors associated with the circumferential widths of RMvI and CMvD and the rate of RNFL thinning were assessed by regression analysis, first with a univariable model and subsequently with a multivariable model that included variables with *P* values < 0.10 on univariate analyses. All statistical analyses were performed using the Statistical Package for the Social Sciences (version 22.0, SPSS, Chicago, IL, USA). Except where indicated otherwise, all data are presented as mean ± SD, with *P* values < 0.05 considered statistically significant.

## Data Availability

The datasets analyzed during the current study available from the corresponding author on reasonable request. The data are not publicly available due to privacy and ethical issue.

## References

[1] Hayreh SS (2001). Blood flow in the optic nerve head and factors that may influence it. Prog. Retin Eye Res..

[2] Flammer J, Orgul S (1998). Optic nerve blood-flow abnormalities in glaucoma. Prog. Retin Eye Res..

[3] Yamamoto T, Kitazawa Y (1998). Vascular pathogenesis of normal-tension glaucoma: a possible pathogenetic factor, other than intraocular pressure, of glaucomatous optic neuropathy. Prog. Retin Eye Res..

[4] Lee EJ, Lee KM, Lee SH, Kim TW (2017). Parapapillary choroidal microvasculature dropout in glaucoma: A comparison between optical coherence tomography angiography and indocyanine green angiography. Ophthalmology.

[5] Suh MH (2016). Deep retinal layer microvasculature dropout detected by the optical coherence tomography angiography in glaucoma. Ophthalmology.

[6] Lee EJ, Kim J-A, Kim T-W (2020). Influence of choroidal microvasculature dropout on the rate of glaucomatous progression: A prospective study. Ophthalmol. Glaucoma.

[7] Park HL, Kim JW, Park CK (2018). Choroidal microvasculature dropout is associated with progressive retinal nerve fiber layer thinning in glaucoma with disc hemorrhage. Ophthalmology.

[8] Kwon JM, Weinreb RN, Zangwill LM, Suh MH (2019). Parapapillary deep-layer microvasculature dropout and visual field progression in glaucoma. Am. J. Ophthalmol..

[9] Kim J-A, Lee EJ, Kim T-W (2019). Evaluation of parapapillary choroidal microvasculature dropout and progressive retinal nerve fiber layer thinning in patients with glaucoma. JAMA Ophthalmol..

[10] Hayreh SS (1969). Blood supply of the optic nerve head and its role in optic atrophy, glaucoma, and oedema of the optic disc. Br. J. Ophthalmol..

[11] Akagi T (2016). Microvascular density in glaucomatous eyes with hemifield visual field defects: An optical coherence tomography angiography study. Am. J. Ophthalmol..

[12] Liu L (2015). Optical coherence tomography angiography of the peripapillary retina in glaucoma. JAMA Ophthalmol..

[13] Lee EJ, Lee KM, Lee SH, Kim TW (2016). OCT Angiography of the peripapillary retina in primary open-angle glaucoma. Invest. Ophthalmol. Vis. Sci..

[14] Yu PK, Cringle SJ, Yu DY (2014). Correlation between the radial peripapillary capillaries and the retinal nerve fibre layer in the normal human retina. Exp. Eye Res..

[15] Lee EJ (2020). Glaucoma-like parapapillary choroidal microvasculature dropout in patients with compressive optic neuropathy. Ophthalmology.

[16] Kwon JM, Weinreb RN, Zangwill LM, Suh MH (2021). Juxtapapillary deep-layer microvasculature dropout and retinal nerve fiber layer thinning in glaucoma. Am. J. Ophthalmol..

[17] Maday S, Twelvetrees AE, Moughamian AJ, Holzbaur EL (2014). Axonal transport: cargo-specific mechanisms of motility and regulation. Neuron.

[18] Lee JY, Shin JW, Song MK, Hong JW, Kook MS (2021). An increased choroidal microvasculature dropout size is associated with progressive visual field loss in open-angle glaucoma. Am. J. Ophthalmol..

[19] Lee J, Park CK, Park H-YL (2021). Determinants of vessel defects in superficial and deep vascular layers in normal-tension glaucoma using optical coherence tomography angiography. Sci. Rep..

[20] The AGIS Investigators. The Advanced Glaucoma Intervention Study (AGIS): 7. The relationship between control of intraocular pressure and visual field deterioration.The AGIS Investigators. *Am. J. Ophthalmol.* **130**, 429–440 (2000). 10.1016/s0002-9394(00)00538-9 11024415

[21] Leske MC (2003). Factors for glaucoma progression and the effect of treatment: the early manifest glaucoma trial. Arch. Ophthalmol..

[22] Group., C. N.-T. G. S. The effectiveness of intraocular pressure reduction in the treatment of normal-tension glaucoma. *Am. J. Ophthalmol.* **126**, 498–505 (1998). 10.1016/s0002-9394(98)00272-4 9780094

[23] Kass, M. A. *et al.* The Ocular Hypertension Treatment Study: a randomized trial determines that topical ocular hypotensive medication delays or prevents the onset of primary open-angle glaucoma. *Arch. Ophthalmol.* **120**, 701–713; discussion 829–730 (2002). 10.1001/archopht.120.6.701 12049574

[24] Heijl A (2002). Reduction of intraocular pressure and glaucoma progression: Results from the Early Manifest Glaucoma Trial. Arch. Ophthalmol..

[25] Collaborative Normal-Tension Glaucoma Study Group. Comparison of glaucomatous progression between untreated patients with normal-tension glaucoma and patients with therapeutically reduced intraocular pressures. Collaborative Normal-Tension Glaucoma Study Group. *Am. J. Ophthalmol.* **126**, 487–497 (1998). 10.1016/s0002-9394(98)00223-2 9780093

[26] Huber K, Plange N, Remky A, Arend O (2004). Comparison of colour Doppler imaging and retinal scanning laser fluorescein angiography in healthy volunteers and normal pressure glaucoma patients. Acta Ophthalmol. Scand..

[27] Findl O (2000). Assessment of optic disk blood flow in patients with open-angle glaucoma. Am. J. Ophthalmol..

[28] Shiga Y (2013). Waveform analysis of ocular blood flow and the early detection of normal tension glaucoma. Invest. Ophthalmol. Vis. Sci..

[29] Shiga Y (2016). Optic nerve head blood flow, as measured by laser speckle flowgraphy, is significantly reduced in preperimetric glaucoma. Curr. Eye Res..

[30] Sehi M (2014). Retinal blood flow in glaucomatous eyes with single-hemifield damage. Ophthalmology.

[31] Schwartz B, Rieser JC, Fishbein SL (1977). Fluorescein angiographic defects of the optic disc in glaucoma. Arch. Ophthalmol..

[32] Hitchings RA, Spaeth GL (1977). Fluorescein angiography in chronic simple and low-tension glaucoma. Br. J. Ophthalmol..

[33] Yamazaki S, Inoue Y, Yoshikawa K (1996). Peripapillary fluorescein angiographic findings in primary open angle glaucoma. Br. J. Ophthalmol..

[34] Laatikainen L (1971). Fluorescein angiographic studies of the peripapillary and perilimbal regions in simple, capsular and low-tension glaucoma. Acta Ophthalmol. Suppl..

[35] O'Brart, D. P., de Souza Lima, M., Bartsch, D. U., Freeman, W. & Weinreb, R. N. Indocyanine green angiography of the peripapillary region in glaucomatous eyes by confocal scanning laser ophthalmoscopy. *Am. J. Ophthalmol.* **123**, 657–666 (1997). 10.1016/s0002-9394(14)71078-5 9152071

[36] Funaki S, Shirakashi M, Abe H (1997). Parapapillary chorioretinal atrophy and parapapillary avascular area in glaucoma. Nippon Ganka Gakkai Zasshi.

[37] Jammal AA (2022). Blood pressure and glaucomatous progression in a large clinical population. Ophthalmology.

[38] Akagi T (2017). Rates of local retinal nerve fiber layer thinning before and after disc hemorrhage in glaucoma. Ophthalmology.

[39] Lee EJ, Kim T-W, Kim J-A, Kim J-A (2017). Parapapillary Deep-Layer microvasculature dropout in primary open-angle glaucoma eyes with a parapapillary γ-Zone. Invest. Ophthalmol. Vis. Sci..

[40] Jonas JB, Papastathopoulos KI (1996). Optic disc shape in glaucoma. Graefes. Arch. Clin. Exp. Ophthalmol..

[41] Vongphanit J, Mitchell P, Wang JJ (2002). Population prevalence of tilted optic disks and the relationship of this sign to refractive error. Am. J. Ophthalmol..

[42] Bengtsson, B., Leske, M. C., Yang, Z., Heijl, A. & group, E. Disc hemorrhages and treatment in the early manifest glaucoma trial. *Ophthalmology* **115**, 2044–2048 (2008). 10.1016/j.ophtha.2008.05.031 18692244

[43] Lee EJ, Kim TW, Kim JA, Kim JA (2018). Central visual field damage and parapapillary choroidal microvasculature dropout in primary open-angle glaucoma. Ophthalmology.

[44] Lee EJ, Kim TW, Lee SH, Kim JA (2017). Underlying microstructure of parapapillary deep-layer capillary dropout identified by optical coherence tomography angiography. Invest. Ophthalmol. Vis. Sci..

[45] Liu, Y. *et al.* Patient characteristics associated with artifacts in Spectralis optical coherence tomography imaging of the retinal nerve fiber layer in glaucoma. *Am. J. Ophthalmol.* **159**, 565–576 (2015). 10.1016/j.ajo.2014.12.006 PMC4423408 25498118

[46] Lee SH, Lee EJ, Kim TW (2018). Topographic correlation between juxtapapillary choroidal thickness and parapapillary deep-layer microvasculature dropout in primary open-angle glaucoma. Br. J. Ophthalmol..

[47] Lee KM, Lee EJ, Kim TW (2016). Juxtapapillary choroid is thinner in normal-tension glaucoma than in healthy eyes. Acta Ophthalmol..

